# Oxygen therapy may worsen the survival rate in rats with monocrotaline-induced pulmonary arterial hypertension

**DOI:** 10.1371/journal.pone.0204254

**Published:** 2018-09-20

**Authors:** Naoto Fujita, Natsuki Yamasaki, Kanako Eto, Makoto Asaeda, Wataru Kuwahara, Hidetaka Imagita

**Affiliations:** 1 Department of Musculoskeletal Functional Research and Regeneration, Graduate School of Biomedicine and Health Sciences, Hiroshima University, Kasumi, Minami-ku, Hiroshima, Japan; 2 Sports Medical Center, Hiroshima University Hospital, Kasumi, Minami-ku, Hiroshima, Japan; 3 Graduate School of Health Sciences, Kio University, Umaminaka, Koryo-cho, Kitakatsuragi-gun, Nara, Japan; Max Delbruck Centrum fur Molekulare Medizin Berlin Buch, GERMANY

## Abstract

Although oxygen therapy rapidly improves arterial oxygen saturation in idiopathic pulmonary arterial hypertension, the effects of chronic administration of oxygen are unknown. The purpose of the present study was to investigate the effects of chronic oxygen therapy on the histological changes and survival rate in rats with idiopathic pulmonary arterial hypertension. Idiopathic pulmonary arterial hypertension was induced by monocrotaline injection. The rats were then randomly assigned to receive or not receive oxygen therapy (O_2_ group and non-O_2_ group, respectively). The rats in the O_2_ group were exposed to a high (90%) oxygen environment from day 17 following injection of monocrotaline, when hypoxemia was first observed. The pulmonary arteriole walls were significantly thicker in monocrotaline-injected rats than in saline-injected rats as vehicle on day 19 and were significantly thicker in the rats that received oxygen therapy than in the rats that did not. Right ventricular inflammations were significantly higher in monocrotaline-injected rats than in saline-injected rats on day 19 and were significantly higher in the rats that received oxygen therapy than in the rats that did not. By day 20 after injection of monocrotaline, the survival rate was significantly lower in the rats that received oxygen therapy than in those that did not. Superoxide dismutase activity in the lungs was higher in monocrotaline-injected rats than in saline-injected rats on day 19 after monocrotaline injection and was also higher in the saline-injected rats that received oxygen therapy than in the saline-injected rats that did not. No interaction was detected between monocrotaline injection and oxygen therapy. These results suggest that chronic oxygen therapy worsens the histological changes and survival rate in idiopathic pulmonary arterial hypertension. The fact that degradation of the histological changes and survival rate was accompanied by increase in superoxide dismutase activity suggests that antioxidant capacity may contribute to the degradation.

## Introduction

Pulmonary arterial hypertension is characterized by progressive histological changes, including medial hypertrophy, intimal thickening, plexiform lesions, dilated lesions, and arteritis, in the pulmonary arteries and arterioles [1] that increase pulmonary arterial blood pressure. These histological changes cause narrowing of the luminal diameter of the pulmonary arteries and arterioles and decreased circulation of blood in the lungs. The disturbance in the pulmonary circulation causes symptoms of dyspnea on effort, shortness of breath at rest, tiredness, and palpitation [2,3,4,5].

Medical therapy, including vasodilators and anticoagulants, is used to treat pulmonary arterial hypertension, and oxygen therapy is also used in patients who develop hypoxemia [2,3,4]. Although oxygen therapy is often used to alleviate dyspnea, there is insufficient knowledge of the therapy for pulmonary arterial hypertension. In contrast, there is some evidence for oxygen therapy on chronic obstructive pulmonary disease (COPD)-related pulmonary hypertension [6,7,8]. Whole day oxygen therapy improved the hemodynamic abnormalities of patients with hypoxic COPD [9]. The efficacy of oxygen therapy in COPD depends on severity of pulmonary arterial hypertension, suggesting that early treatment would improve the prognosis [10]. Currently, although the pathogenic mechanisms differ between idiopathic pulmonary arterial hypertension and COPD-related pulmonary hypertension, oxygen therapy is performed based on limited knowledge of the therapy for COPD.

Roberts *et al*. reported that 100% oxygen rapidly improves arterial oxygen saturation and pulmonary vascular resistance and decreases mean pulmonary artery pressure in patients with pulmonary arterial hypertension [11]. However, there is no clear evidence that chronic oxygen therapy improves pulmonary arterial hypertension. Weitzenblum *et al*. reported that chronic oxygen therapy decreased pulmonary artery pressure in patients with pulmonary hypertension associated with COPD [12]. However, the effects of longer-term oxygen therapy in patients with idiopathic pulmonary arterial hypertension are still unknown. Histological changes, such as medial hypertrophy in the pulmonary arteries and arterioles, is milder in patients with COPD-related pulmonary hypertension than in those with idiopathic pulmonary arterial hypertension [13]. Therefore, the effects of chronic oxygen therapy in pulmonary hypertension associated with COPD would not necessarily be consistent with those in idiopathic pulmonary arterial hypertension. The purpose of the present study was to investigate the effects of chronic oxygen therapy on the histological changes and survival rate in rats with idiopathic pulmonary arterial hypertension.

## Materials and methods

### Experimental design

The study was approved by the Institutional Animal Care and Use Committee of Hiroshima University (A13-30), and was carried out according to the Hiroshima University Regulations for Animal Experimentation. All experiments were conducted in accordance with the National Institute of Health (NIH) Guidelines for the Care and Use of Laboratory Animals (National Research Council, 1996).

Eighteen 4-week-old male Wistar rats (mean weight 89 ± 3 g) were used to investigate the survival rate. The rats having a body weight of 85–100 g were selected to induce pulmonary arterial hypertension based on the previous study [14]. All the rats received a single intraperitoneal injection of monocrotaline (30 mg/kg) to induce pulmonary arterial hypertension with subsequent right ventricular hypertrophy and failure. It is known that monocrotaline is metabolized and changed to the active form in the liver, after which it injures the vascular endothelium of the pulmonary vessels and causes pulmonary arterial hypertension [15]. In our previous study, cachexia induced by right heart failure was confirmed at 21 days after an equivalent dose of monocrotaline [16]. Furthermore, light dyspnea was found at 17 days after the monocrotaline injection in a preliminary study (data not shown). Therefore, 17 days after the monocrotaline injection, the rats were randomly assigned to receive oxygen therapy (O_2_ group, n = 9) or not to receive oxygen therapy (non-O_2_ group, n = 9). Rats in the O_2_ group were exposed to a high (90%) oxygen environment that was continued uninterrupted for 4 days with the exception of breaks for daily cage cleaning. The rats in the non-O_2_ group were housed in normal air (21% oxygen). Heart rate, arterial oxygen saturation, and respiratory rate were measured by pulse oximeter (STARR Life Sciences, Oakmont, PA, USA) for the duration of oxygen therapy, i.e., 17–21 days after monocrotaline injection. The health of the rats was monitored twice a day. If the rats expressed the specific signs such as severe weight loss, tachypnea, and lethargy, we judged the signs as terminal. The rats that showed the signs (*i*.*e*., rats nearing death) were humanely euthanized by an overdose of sodium pentobarbital to minimize the distress. Six age-matched male Wistar rats (mean weight 87 ± 4 g) were used to determine the influence of expose to high oxygen in normal animals. These rats were exposed to 90% oxygen or normal air after 17 days of injection of an equivalent volume of saline (saline O_2_ group, saline non-O_2_ group; n = 3 in each group), and their body weight was measured daily. All rats were housed in a controlled environment on a fixed 12-h light-dark cycle and at a constant temperature of 22 ± 2°C. Food and water were provided ad libitum.

Eighteen age-matched male Wistar rats (mean weight 89 ± 4 g) were used for retrieval of the lungs and heart. These rats were randomly assigned to receive an injection of monocrotaline (MCT group, n = 12) or an equivalent volume of saline (saline group, n = 6). These rats were randomly allocated to receive or not receive oxygen therapy (MCT O_2_ group, MCT non-O_2_ group [n = 6 in each group]; saline O_2_ group, saline non-O_2_ group [n = 3 in each group]). The six rats in the MCT O_2_ and MCT non-O_2_ groups (n = 3 in each group) were euthanized by an overdose of sodium pentobarbital on day 17 after monocrotaline injection. The samples collected on day 17 after monocrotaline injection were used to confirm pulmonary vascular remodeling before initiation of oxygen therapy. The 12 rats (MCT O_2_ group, MCT non-O_2_ group, saline O_2_ group, saline non-O_2_ group; n = 3 in each group) were euthanized by an overdose of sodium pentobarbital on day 19 after monocrotaline injection (*i*.*e*., 2 days after initiation of oxygen therapy). Because inflammation generally peaks 48 h after acute stress, the samples collected after 2 days of oxygen therapy were used to detect inflammatory changes. The heart and right lung were removed immediately from each rat, frozen in liquid nitrogen, and stored at -80°C until analysis. The left lung was subsequently removed, and the airways were filled with 4% paraformaldehyde in 0.1 M phosphate buffer, and embedded in paraffin.

### Histological analysis

Transverse sections were obtained from the left lung using a microtome. The sections were stained with Elastica van Gieson to calculate the relative wall thickness of the pulmonary arterioles. At least 30 pulmonary arterioles with an outer medial wall diameter around 50 μm were randomly chosen for each rat. Wall thickness was calculated as the ratio of the medial wall diameter to the external wall diameter based on the previous study [17]. The calculations were performed in a blinded fashion.

Transverse sections were obtained from the right ventricle using a cryostat microtome, fixed in -20°C acetone, and blocked with Blocking One Histo (Nakalai Tesque, Kyoto, Japan). The sections were incubated at 4°C overnight with anti-dystrophin (1:200, sc-15376; Santa Cruz Biotechnology, Dallas, TX, USA) to identify shape of cardiocyte and anti-CD45 (1:100, sc-53045; Santa Cruz Biotechnology) antibodies to identify infiltration of leukocytes into the right ventricle. The sections were then exposed to Alexa Fluor 488 or 555 conjugated anti-rabbit or anti-mouse immunoglobulin G (1:1000; Cell Signaling Technology, Danvers, MA, USA) for 60 min at room temperature, and mounted with medium containing 4′,6-diamidino-2-phenylindole to identify nuclei (H-1500, Vector Laboratories, Orton Southgate, UK). Parallel slides without the primary antibody were processed identically and served as negative controls. The sections were analyzed and images were acquired using a fluorescence microscope (BZ-9000, Keyence, Osaka, Japan). These sections were used to measure the cross-sectional area of the cardiomyocytes. Cardiomyocytes (at least 50 per animal) that were close in shape to a perfect circle were used when evaluating the cross-sectional area. The number of CD45-positive nuclei was quantified in three fields for each rat. All measurements were carried out using ImageJ software (National Institutes of Health, Bethesda, MD, USA).

### Analysis of superoxide dismutase activity

The right lung samples were homogenized in 10 mM Tris-HCl buffer (pH 7.4) containing 0.25 M sucrose and 1 mM ethylenediaminetetraacetic acid. After centrifugation at 10,000 × g for 60 min at room temperature, the supernatants were collected. After measurement of total protein concentration, superoxide dismutase (SOD) activity was measured by spectrophotometry using a commercial kit (S311; Dojindo Molecular Technologies, Kumamoto, Japan) according to the manufacturer’s instructions.

### Analysis of protein carbonyl

The right lung samples were homogenized in phosphate buffered saline (pH 7.4) containing 1% protease inhibitor cocktail (25955–11; Nakalai Tesque). After centrifugation at 12,000 × g for 10 min at 4°C, the supernatants were collected. After measurement of total protein concentration, protein carbonyl was measured using a commercially available enzyme-linked immunosorbent assay kit (STA-310; Cell biolabs, CA, USA) according to the manufacturer’s instructions.

### Quantitative polymerase chain reaction (qPCR) analysis

Total RNA was isolated from right lung sample using TRIzol reagent (15596–026; Invitrogen, Tokyo, Japan). Reverse transcription was performed using the High-Capacity cDNA Reverse Transcription Kit (4374966; Applied Biosystems, Foster City, CA, USA), and the resultant cDNA samples were stored at −20°C. The expression levels of nicotinamide adenine dinucleotide phosphate oxidase (Nox) 1 (Rn00586652_m1) and Nox2 (Rn00576710_m1) mRNA were quantified using qPCR with TaqMan Gene Expression Assays (Applied Biosystems). The relative expression levels of Nox1 and Nox2 were inferred by normalizing the quantity of cDNA template for each gene against the quantity of cDNA for the normalization gene 18S (Rn03928990_g1). The cDNA concentration at each qPCR cycle was plotted to obtain a standard curve, and the relative gene expression was obtained as the value at the threshold line. qPCR was performed in CFX96 (Bio-Rad Laboratories, Hercules, CA, USA) using the PCR Fast Advanced Master Mix (Applied Biosystems).

### Statistical analysis

The data are reported as the mean ± standard deviation. The statistical significance of differences between groups was determined by the independent *t*-test or two-way analysis of variance. Statistical significance was set at *p* < 0.05.

## Results

### Arterial oxygen saturation

**Fig 1:**
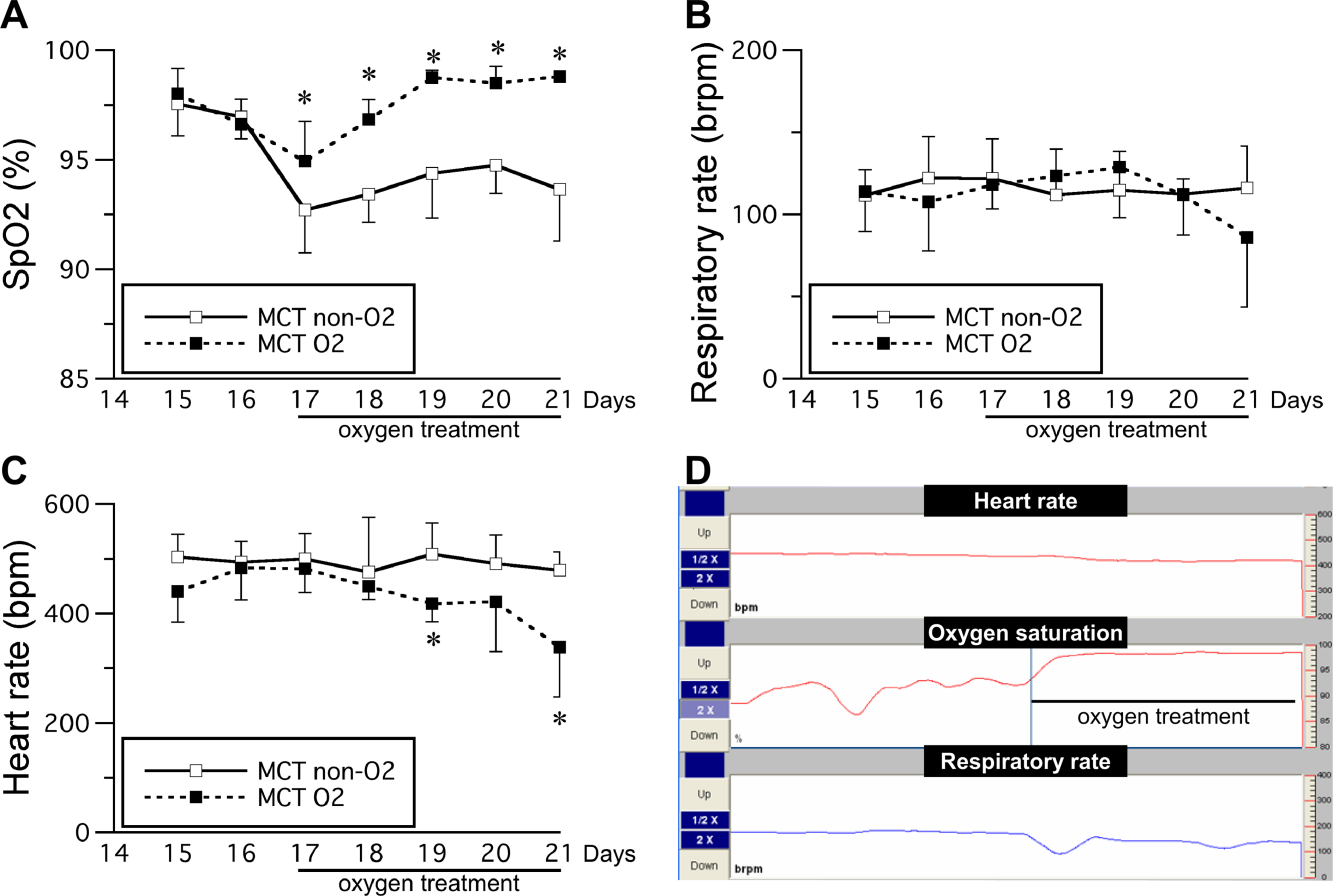
**Time course of changes in oxygen saturation (A), respiratory rate (B), and heart rate (C) after monocrotaline injection and representative data during oxygen therapy (D).** Oxygen therapy was initiated 17 days after monocrotaline injection. MCT O_2_, monocrotaline injection with oxygen therapy (total n = 9; 15–19 days, n = 9; 20 day, n = 7; 21 day, n = 2); MCT non-O_2_, monocrotaline injection without oxygen therapy (total n = 9; 15–17 days, n = 9; 18–21 days, n = 8). The data are shown as the mean ± standard deviation. *Significantly different from the MCT non-O_2_ group by independent *t*-test, *p* < 0.05.

Oxygen saturation had decreased to below 95% in the MCT non-O_2_ group by 17 days after monocrotaline injection (Fig 1A). However, in the MCT O_2_ group, oxygen saturation was immediately stabilized by oxygen therapy and remained above 95%. Oxygen saturation was significantly higher in the MCT O_2_ group than in the MCT non-O_2_ group on day 17 after injection of monocrotaline. There was no significant difference in respiratory rate between the MCT non-O_2_ and MCT O_2_ groups (Fig 1B). Heart rate was significantly lower in the MCT O_2_ group than in the MCT non-O_2_ group on days 19 and 21 after injection of monocrotaline (Fig 1C). Although the heart rate and respiratory rate were slightly decreased following oxygen therapy, there was no severe respiratory depression during treatment (Fig 1D).

### Survival rate and body weight

**Fig 2:**
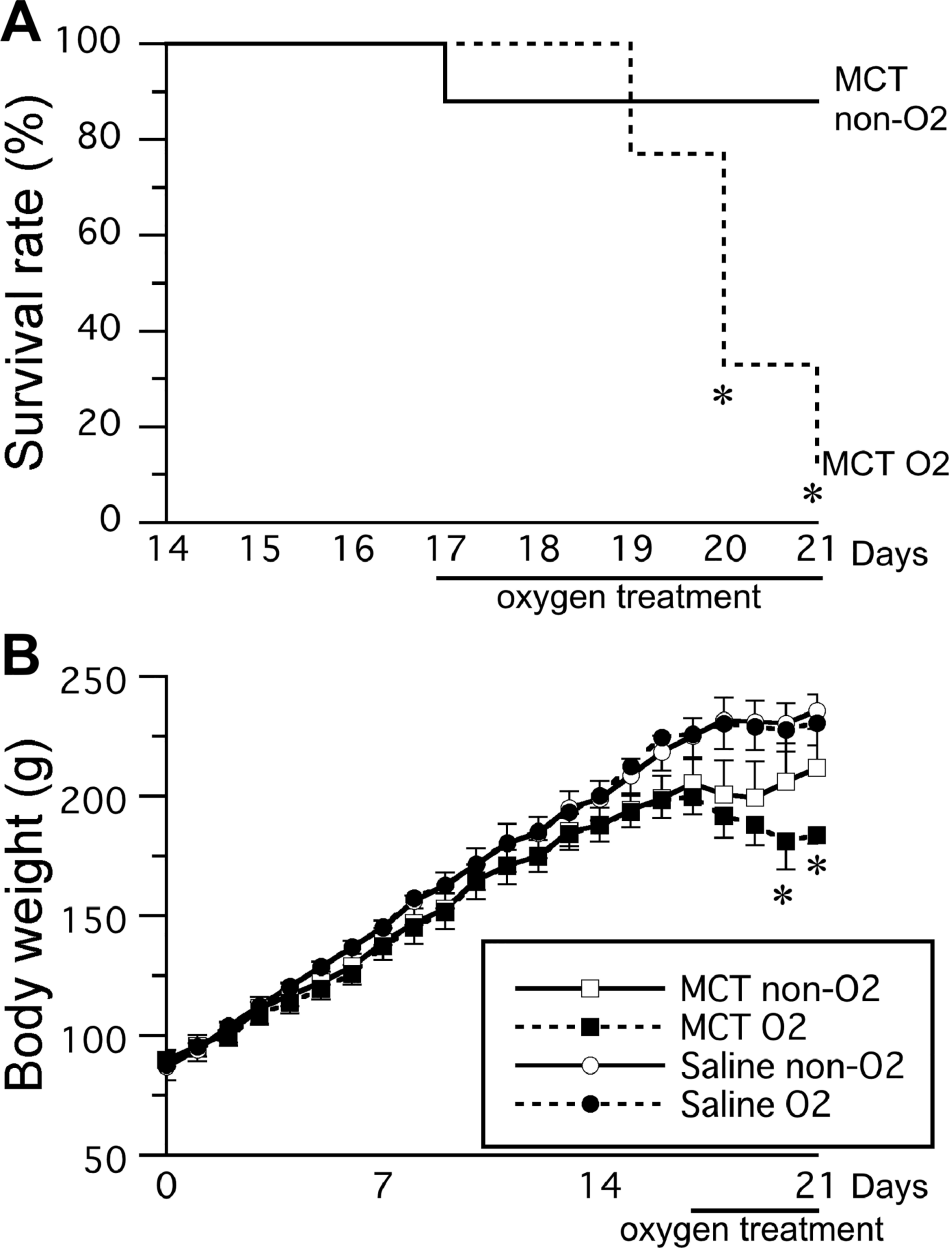
**Survival rate (A) and body weight (B) after monocrotaline injection.** Oxygen therapy was initiated 17 days after monocrotaline injection. Saline O_2_, saline injection with oxygen therapy (n = 3); Saline non-O_2_, saline injection without oxygen therapy (n = 3). MCT O_2_ and MCT non-O_2_ groups: n = 9 in each group. The data are shown as the mean ± standard deviation. *Significantly different from the MCT non-O_2_ group by independent *t*-test, *p* < 0.05.

One rat in the MCT non-O_2_ group and eight rats in the MCT O_2_ group died during the study period (Fig 2A). The survival rate decreased markedly on day 19 after injection of monocrotaline (*i*.*e*., after 2 days of oxygen therapy). The survival rate was significantly lower in the MCT O_2_ group than in the MCT non-O_2_ group on day 20 after monocrotaline injection.

There was a daily increase in body weight in both the MCT O_2_ group and the MCT non-O_2_ group until day 17 after monocrotaline injection; thereafter, body weight decreased, particularly in the MCT O_2_ group (Fig 2B). Body weight was significantly lower in the MCT O_2_ group than in the MCT non-O_2_ group on day 20 after monocrotaline injection. There was no significant difference in body weight on day 20 between the saline O_2_ group and the saline non-O_2_ group.

### Pulmonary vascular remodeling

**Fig 3:**
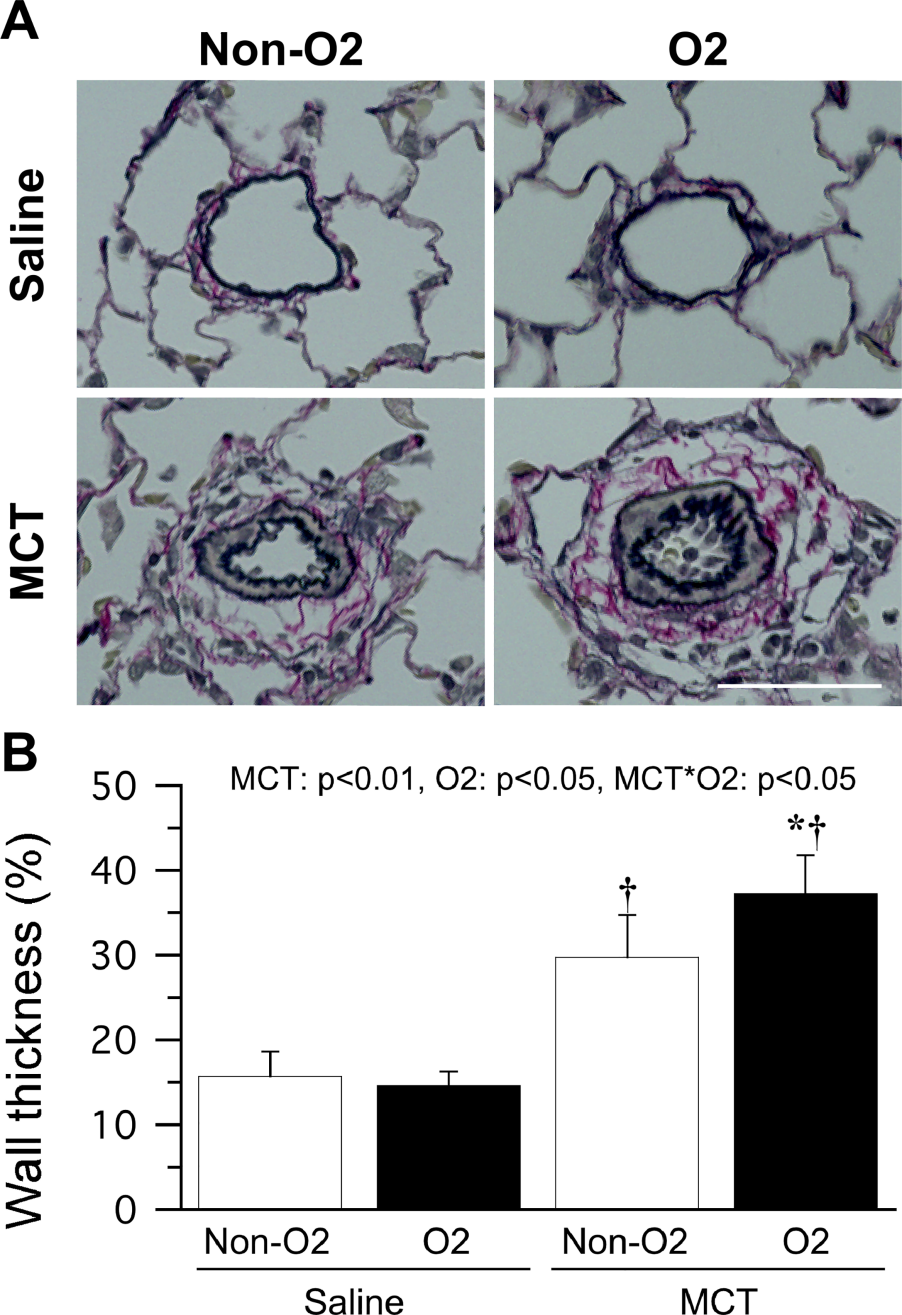
Pulmonary vascular remodeling after 2 days of oxygen therapy. Representative transverse sections of pulmonary arterioles stained with Elastica van Gieson (A). MCT, monocrotaline injection group; Saline, saline injection group; O_2_, oxygen therapy group; non-O_2_, non-oxygen therapy group. Bar = 50 μm. Pulmonary arteriole wall thickness (B). n = 3 in each subgroup. The data are shown as the mean ± standard deviation. MCT, O2, MCT × O2 denote main effects of monocrotaline injection, oxygen therapy, and interaction between monocrotaline injection and oxygen therapy by two-way analysis of variance, respectively. * and † indicate a significant difference when compared with the MCT non-O_2_ group and saline group, respectively.

Monocrotaline injection had induced medial wall thickening in the pulmonary arteries, particularly in the MCT O_2_ group, by day 19 after monocrotaline injection (*i*.*e*., after 2 days of oxygen therapy, Fig 3A). The walls of the pulmonary arterioles were significantly thicker in the MCT group than in the saline group (Fig 3B) and were also significantly thicker in the MCT O_2_ group than in the MCT non-O_2_ group. For the samples collected on day 17 after monocrotaline injection, pulmonary vascular remodeling was confirmed before initiation of oxygen therapy. There was no significant difference in pulmonary arteriole wall thickness between days 17 (27 ± 4%) and 19 (29 ± 4%) after monocrotaline injection (*i*.*e*., before and after 2 days of oxygen therapy) in the MCT non-O_2_ group. However, the pulmonary arteriole wall thickness was significantly greater on day 19 (37 ± 4%) than on day 17 (31 ± 9%) in the MCT O_2_ group.

**Fig 4:**
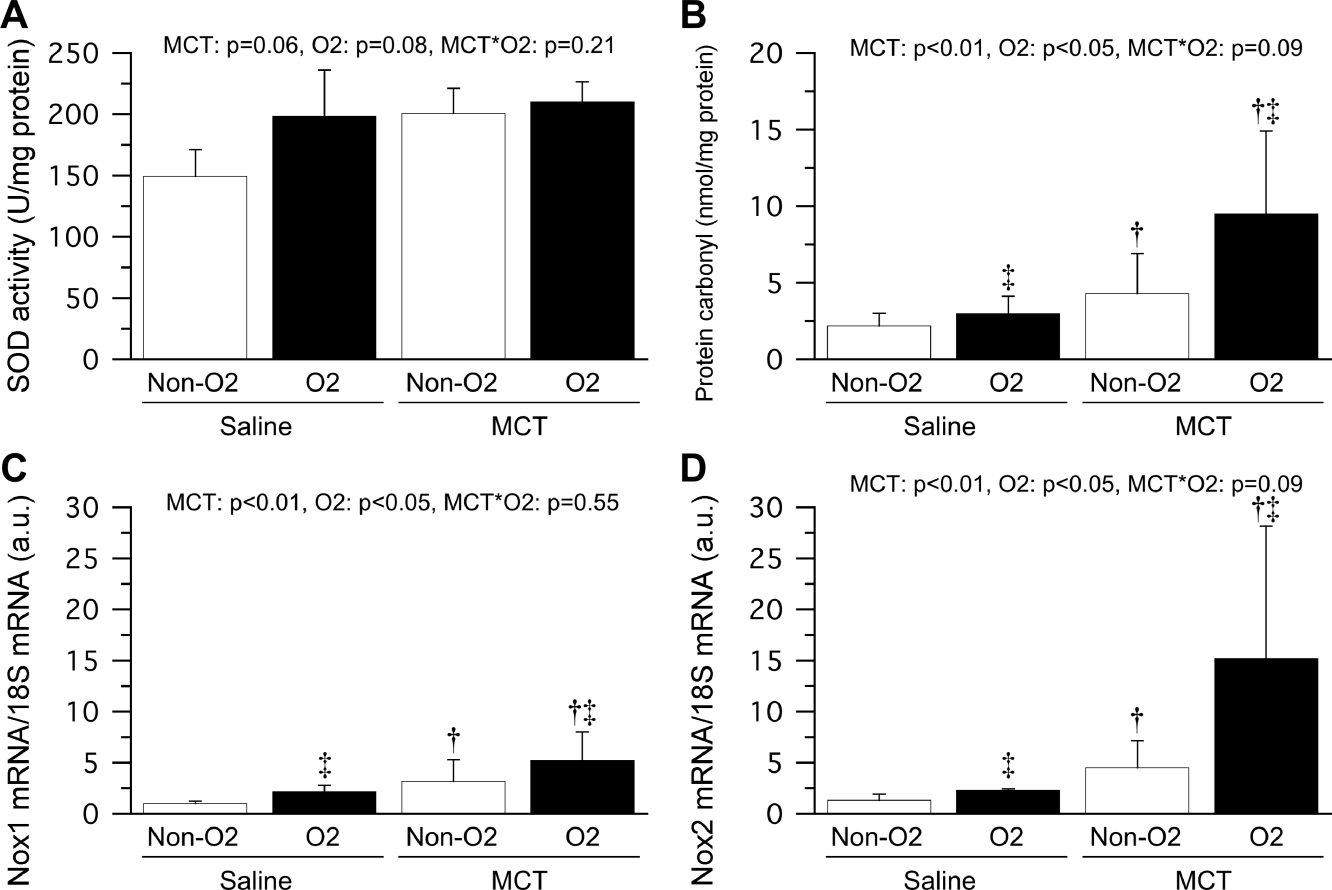
Oxidative stress and antioxidant capacity in the lung. Superoxide dismutase (SOD) activity (A), protein carbonyl content (B), expression level of nicotinamide adenine dinucleotide phosphate oxidase (Nox) 1 (C), and Nox2 (D) mRNA. MCT, monocrotaline injection group; Saline, saline injection group; O_2_, oxygen therapy group; non-O_2_, non-oxygen therapy group. n = 3 in each subgroup. The data are shown as the mean ± standard deviation. MCT, O2, MCT × O2 denote main effects of monocrotaline injection, oxygen therapy, and interaction between monocrotaline injection and oxygen therapy by two-way analysis of variance, respectively. † and ‡ indicate a significant difference when compared with the saline group and non-O_2_ group, respectively.

SOD activity tended to be higher in the MCT group than in the saline group and to be higher in the O_2_ group than in the non-O_2_ group (Fig 4A). Protein carbonyl contents were significantly higher in the MCT group than in the saline group, and significantly higher in the O_2_ group than in the non-O_2_ group (Fig 4B). However, in SOD activity and protein carbonyl contents, there was no interaction between monocrotaline injection and oxygen therapy. The expression levels of Nox1 (Fig 4C) and Nox2 (Fig 4D) mRNA were significantly higher in the MCT group than in the saline group, and significantly higher in the O_2_ group than in the non-O_2_ group. However, there was no interaction between monocrotaline injection and oxygen therapy.

### Cardiac inflammation

**Fig 5:**
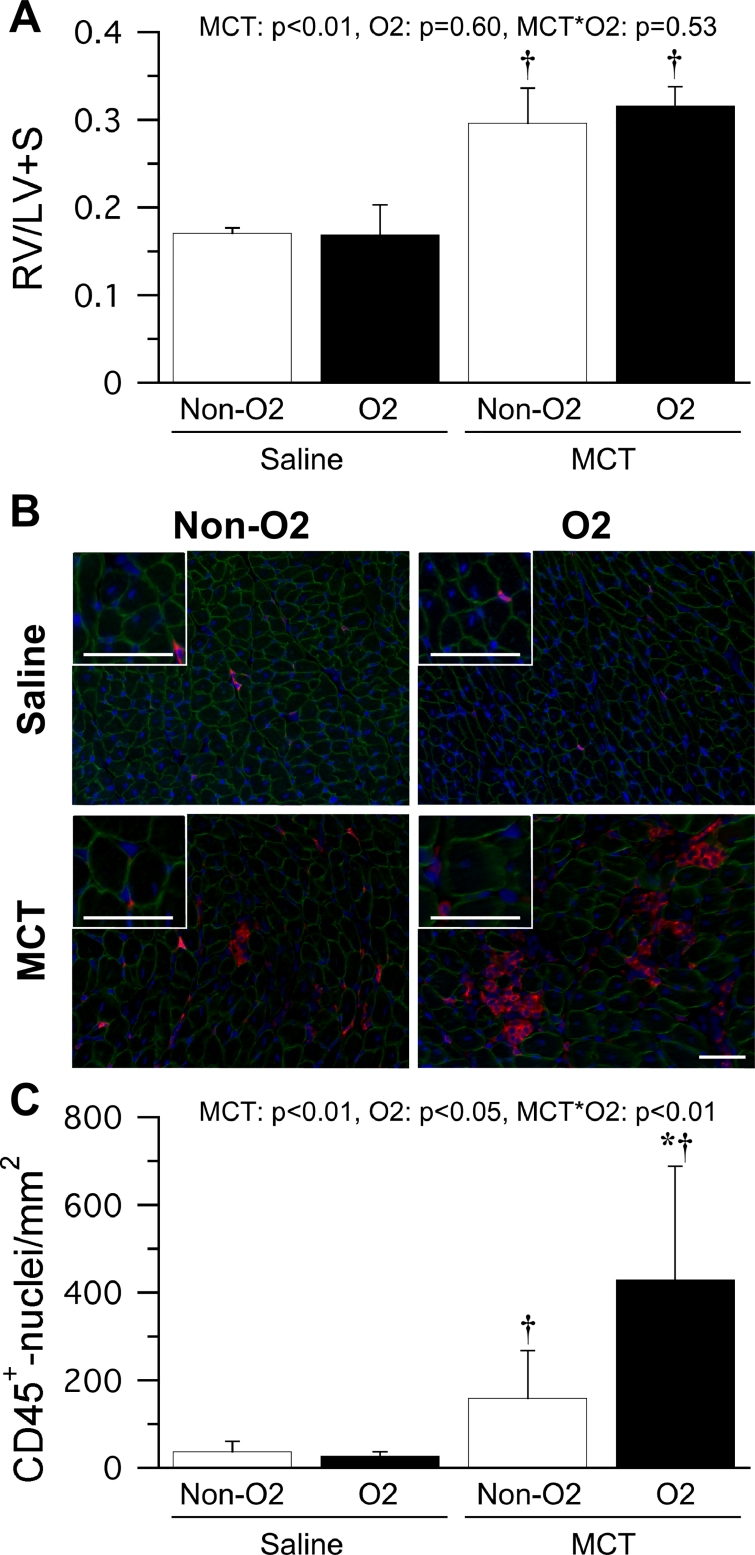
Right ventricular inflammation at 2 days after oxygen therapy. The ratio of right ventricle to left ventricle and septum weights (A). RV, right ventricle; LV, left ventricle, S, septum. Representative merged images (B) of signals for dystrophin (green), CD45 (red), and 4′,6-diamidino-2-phenylindole (blue). Bar = 50 μm. Number of CD45-positive nuclei detected in the right ventricle (C). n = 3 in each subgroup. The data are shown as the mean ± standard deviation. MCT, O2, MCT × O2 denote main effects of monocrotaline injection, oxygen therapy, and interaction between monocrotaline injection and oxygen therapy by two-way analysis of variance, respectively. * and † indicate a significant difference when compared with the MCT non-O_2_ group and saline group, respectively.

Fulton index (ratio of right ventricle to left ventricle and septum weights) was significantly higher in the MCT group than in the saline group (Fig 5A). Marked hypertrophy of cardiomyocytes and infiltration of CD45-positive leukocytes into the right ventricle were detected after injection of monocrotaline (Fig 5B), particularly in the MCT O_2_ group. The cross-sectional areas of the cardiomyocytes were 297 ± 23 μm^2^ in the saline non-O_2_ group, 307 ± 24 μm^2^ in the saline O_2_ group, 451 ± 33 μm^2^ in the MCT non-O_2_ group, and 700 ± 66 μm^2^ in the MCT O_2_ group. The cross-sectional cardiomyocyte area was significantly larger in the MCT group than in the saline group and was also significantly larger in the MCT O_2_ group than in the MCT non-O_2_ group. The number of CD45-positive nuclei in the right ventricle was significantly higher in the MCT group than in the saline group (Fig 5C) and was also significantly higher in the MCT O_2_ group than in the MCT non-O_2_ group on day 19 (*i*.*e*., after 2 days of oxygen therapy).

## Discussion

In this study, chronic oxygen therapy prevented a decrease in oxygen saturation in rats with pulmonary arterial hypertension but caused weight loss and decreased the survival rate. We also found that chronic oxygen therapy accelerated medial hypertrophy in the pulmonary arterioles induced by monocrotaline. Chronic oxygen therapy also promoted infiltration of leukocytes into the right ventricle and cardiac inflammation.

Previous studies reported survival rates of 70%–90% in rats in the 21 days following monocrotaline injection [18,19,20]. In the present study, the survival rate in monocrotaline-injected rats that received oxygen therapy was 11%, which is extremely low in comparison with the results of the earlier studies. The severity of pulmonary arterial hypertension induced by monocrotaline depends on the dose administered [17], which was lower (30 mg/kg) in our study than the dose of 60 mg/kg used in the previous studies. Therefore, the low survival rate is attributed to the worsening pulmonary arterial hypertension resulting from chronic oxygen therapy.

The effect of oxygen therapy could depend on the pathogenesis of pulmonary arterial hypertension. Some positive effects have been reported in secondary pulmonary hypertension with COPD [8,21,22]. However, thus far, there have been no controlled studies of the effects of longer-term oxygen therapy in idiopathic pulmonary arterial hypertension. The only relevant publication is a single case report [23]. Furthermore, nocturnal oxygen therapy does not alter the survival rate in patients with advanced Eisenmenger syndrome and pulmonary hypertension [24]. Therefore, use of chronic oxygen therapy is currently based on the evidence in COPD [25], in which the pathology in the pulmonary arteries and arterioles is milder than that in idiopathic pulmonary arterial hypertension [13]. Overall, the efficacy of oxygen therapy is likely to depend on the pathogenesis and severity of pulmonary arterial hypertension.

Pulmonary vascular remodeling has been considered a primary development in idiopathic pulmonary arterial hypertension and a secondary development in COPD-related pulmonary hypertension [26]. The severity of pulmonary vascular remodeling at the onset of hypoxemia could differ between primary and secondary pulmonary hypertension. In primary pulmonary hypertension, as in the present study, there is decreased arterial oxygen saturation along with medial hypertrophy in the pulmonary arterioles. In contrast, decreased arterial oxygen saturation precedes medial hypertrophy in secondary pulmonary hypertension [27]. Oxygen therapy is often started after onset of hypoxemia, so the pathology of the pulmonary vessels could be more severe in primary pulmonary hypertension than in secondary pulmonary hypertensions at the start of oxygen therapy. Severe pulmonary arterial hypertension and hyperoxia induced by oxygen therapy usually accompanies oxidative stress [28,29]. In the present study, SOD activity and protein carbonyl contents appeared to be increased by monocrotaline injection. Oxygen therapy also appeared to increase SOD activity and protein carbonyl contents. However, interaction between monocrotaline injection and oxygen therapy was not observed. Similarly, the interaction was not observed in both Nox1 and Nox2 that lead to arteriosclerosis and hypertension due to inactivation of nitric oxide by production of superoxide [30]. Therefore, antioxidant capacity could not sufficiently eliminate the oxidative stress caused by the combination of severe pulmonary artery hypertension and high levels of oxygen, which would accelerate pulmonary vascular pathology and induce right heart failure. Moreover, markedly high numbers of hypertrophic cardiomyocytes and CD45-positive leukocytes were observed in the right ventricle in the monocrotaline-injected rats that received oxygen therapy. Increased numbers of CD45-positive leukocytes in the right ventricle of the MCT O2 group compared with the MCT non-O2 group indicate that oxygen therapy could promote inflammation in right heart failure secondary to pulmonary artery hypertension. Although the cross-sectional cardiomyocyte area was significantly larger in the MCT O_2_ group than in the MCT non-O_2_ group, there was no significant difference in the Fulton index between these two groups. We attribute that the lack of a significant difference in the Fulton index between the MCT non-O_2_ and MCT O_2_ groups to inflammation in the samples collected in this study. The images in the MCT O_2_ group contained not only hypertrophic cardiomyocytes but also large areas of necrosis. These necrotic areas might have led to paradoxical results with regard to the cardiomyocyte cross-sectional area and the Fulton index. Acceleration of pulmonary vascular pathology in response to exposure to a high oxygen concentration may induce right heart failure and worsening of inflammation.

The present study revealed that although oxygen therapy rapidly improves arterial oxygen saturation in idiopathic pulmonary arterial hypertension induced by monocrotaline injection, chronic use of oxygen therapy worsens the survival rate and may induce histological changes. However, our study has some limitations. We did not measure right ventricular or pulmonary blood pressure values. Furthermore, our study did not investigate the effects of oxygen concentration and flow volume or the influence of severity of pulmonary vascular remodeling at the start of oxygen therapy on the histological changes. Oxygen therapy has decreased the mortality rate in patients with cor pulmonale associated with COPD [31]. However, oxygen therapy is not able to inhibit progression of respiratory failure in all patients, suggesting that several factors might be involved in its efficacy. There has been a report of decreased mortality in patients with COPD who received oxygen therapy, indicating that this therapy may be of particular benefit in patients with mild stage of pulmonary arterial hypertension and lower pulmonary vascular resistance [32]. Oxygen therapy was started after onset of hypoxemia in the present study, which may influence the results. It is possible that early oxygen therapy before onset of hypoxemia is effective on the histological changes and survival rate, although it is not possible to verify such a conclusion based on the data in the present study. Moreover, we were unable to clarify the pathophysiologic mechanisms involved in the adverse effects of expose to high oxygen in idiopathic pulmonary arterial hypertension. More data are needed to clarify these mechanisms. Further studies with more data and large sample size are needed to determine the effects of chronic oxygen therapy in idiopathic pulmonary arterial hypertension.
